# To be, or not to be? The role of the unconscious in transgender transitioning: identity, autonomy and well-being

**DOI:** 10.1136/medethics-2021-107397

**Published:** 2021-07-30

**Authors:** Alessandra Lemma, Julian Savulescu

**Affiliations:** 1 Visiting Professor, Psychoanalysis Unit, University College London, London, UK; 2 Faculty of Philosophy, University of Oxford, Oxford, UK; 3 Biomedical Ethics Research Group, Murdoch Childrens Research Institute, Parkville, Victoria, Australia; 4 Melbourne Law School, University of Melbourne, Melbourne, Victoria, Australia

**Keywords:** autonomy, children, concept of health, ethics, sexuality/gender

## Abstract

The exponential rise in transgender self-identification invites consideration of what constitutes an ethical response to transgender individuals’ claims about how best to promote their well-being. In this paper, we argue that ‘accepting’ a claim to medical transitioning in order to promote well-being would be in the person’s best interests iff at the point of request the individual is correct in their self-diagnosis as transgender (i.e., the distress felt to reside in the body does not result from another psychological and/or societal problem) such that the medical interventions they are seeking will help them to realise their preferences. If we cannot assume this—and we suggest that we have reasonable grounds to question an unqualified acceptance in some cases—then ‘acceptance’ potentially works against best interests. We propose a distinction between ‘acceptance’ and respectful, in-depth exploration of an individual’s claims about what promotes their well-being. We discuss the ethical relevance of the unconscious mind to considerations of autonomy and consent in working with transgender individuals. An inquisitive stance, we suggest, supports autonomous choice about how to realise an embodied form that sustains well-being by allowing the individual to consider both conscious and unconscious factors shaping wishes and values, hence choices.

## Introduction: definitions and aims

Referrals to gender identity services (GIDs) in the UK have increased exponentially.^1 2^ This increase has exposed the very considerable challenges facing individuals who identify as transgender. It has also raised concern about how the laudable aims of gender affirmative care may be ushering too quickly children and young people into medical transitioning leading subsequently to a wish to detransition with all the attendant physical and psychological complications.

A recent landmark decision in the UK illustrates this. In the UK, treatment for patients under 18 years presenting with gender dysphoria is offered by the Gender Identity Development Service provided by the Tavistock Clinic. In December 2020, a former patient who began taking puberty blockers when she was 16, before subsequently detransitioning, and the parent of a 15-year-old autistic girl who was on the waiting list for treatment, successfully brought a case against the service. The High Court ruling deemed that patients under 16 years should be assumed not to have capacity to consent to such interventions. This was for two reasons: first—and this was the main argument—children who have not yet gone through puberty are not able to properly understand the ‘lifelong medical, psychological and emotional implications’ of taking puberty blockers and cross-sex hormones; and second, the experimental nature of puberty blockers specifically, with potentially significant unknown side effects and little evidence of long-term benefits.^3^ In practice, this ruling now forbids the prescription of puberty suppressants without court order. This particular ruling is in the process of being challenged by the GID. A more recent 2021 court order allows for the prescription as long as there is parental consent. These medical interventions continue to be provided in many other countries. Such cases invite consideration of what constitutes an ethical response to transgender individuals’ consciously stated claims about how best to promote their well-being.

Given the heterogeneity of transgender identities and experience,^4^ it is important to clarify definitions and scope. In this paper, we are focusing only on binary and non-binary transgender individuals who wish to medically transition (via cross-sex hormones and/or sex reassignment surgery) in order to minimise their distress due to the felt incongruence between the natal body (and assigned gender at birth) and the body they believe will be congruent with their gender of (self-) identification. For present purposes, we are not concerned with the group more accurately described as ‘gender non-conforming’ who often only seek partial or no medical transitioning. However, the group who present for medical transitioning will inevitably comprise some gender non-conforming people who see themselves as needing full transitioning. We do not restrict the discussion to a particular age group except where specified. We will examine the extent to which unconscious forces may undermine autonomy and this argument applies both to children and adults.

We argue that ‘accepting’ a claim to medical transitioning in order to promote well-being would be in the person’s best interests iff at the point of request the individual is correct in their self-diagnosis as transgender (i.e., the distress felt to reside in the body does not result from another psychological and/or societal problem) such that the medical interventions they are seeking will help them to realise their preferences. If we cannot assume this—and we suggest that we have reasonable grounds to question an unqualified acceptance in some cases—then ‘acceptance’ potentially works against best interests. This is important because the treating clinician is also required to make a diagnosis of ‘gender dysphoria’, if they operate according to DSM-5 (*Diagnostic and Statistical Manual of Mental Disorders*, 5th Edition), and so the clinician is required to confirm or validate the patient’s self-diagnosis. Such a clinical formulation must take into account unconscious factors as well as that which is consciously articulated by the patient. We propose a distinction between ‘acceptance’ (here used interchangeably with the current mandate in healthcare for a ‘gender affirming’ approach), and respectful, in-depth exploration of an individual’s claims about what promotes their well-being. We discuss the ethical relevance of the unconscious to considerations of autonomy and consent in working with transgender individuals. An inquisitive stance, we suggest, supports autonomous choice about how to realise an embodied form that sustains well-being by allowing the individual to consider both conscious and unconscious factors shaping wishes and values, hence choices.

The relationship between autonomy and best interests (as conceived of as well-being) is a complicated and contested one. One of us (JS) conceives of autonomy as constituted by our rational desires or values.^5^ This is separate to well-being, which can be conceived of either objectively or subjectively. On a purely objective conception of well-being (objective list approach),^6^ well-being and autonomy can come apart. For example, people may autonomously desire their own lesser good, for example, Jehovah’s Witnesses refusing life-saving blood transfusions. Some philosophers include autonomy as one item on an objective list.^6^ On such an account, what a person strongly autonomously desires will be in their interests. Others hold a purely subjective account of well-being which collapses autonomy and well-being (interests).

Fortunately, for this paper, we do not need to resolve this issue. In the case of gender dysphoria, the accuracy of a person’s appreciation of their own self, identity and values (which constitute their autonomy) will be strongly determinative of their interests. For example, one item on an objective list is deep personal relationships.^6 7^ Misunderstanding the nature of one’s own relationships will compromise both autonomy and interests. We will focus on autonomy, which in this case will have implications for the best interests of the person with gender dysphoria.

## The merits and challenges of gender affirmative care

The question about whether we should ‘accept’ transgender individuals’ claims about what will support their well-being can be approached from two angles: one more obvious, generalisable, but no less important and the other, more variegated and hence not allowing any generalisations. We will first address the more obvious reading before exploring a more complex reading of ‘acceptance’.

The etymology of ‘to accept’ is the Latin *accipere* meaning ‘to receive, to let in, admit, hear, learn’.^8^ It denotes a willingness to take on board another person’s account, to give it legitimacy, but not necessarily to accept it as true or superior to an alternative account. Ethical decision-making about any medical intervention that carries risks as well as potential benefits is always embedded in a relational matrix in which at least two people strive to arrive at a decision about whether an intervention is in the patient’s best interests. This is central to most contemporary models of the doctor-patient relationship and represents a rejection of the historically dominant medical model of medical paternalism. Within the 1980s and 90s, respect for patient autonomy increased in importance in medicine. But tit also came with the realisation that medicine is concerned with promoting the patient’s best overall interests, such as well-being, rather than health narrowly conceived as the absence of disease. As medicine became increasingly complex, with multiple options with different risk/benefit profiles, it became clear it was necessary to understand patient values to identify the best option. According to the shared decision-making model, patients supplied the values and doctors supplied the facts. However, other models gave greater weight to normative dialogue, that is, dialogue about should be done, what is good and right. For example, the liberal rationalist model required doctors and patients to engage in normative and value dialogue, as well as exchange of facts, to identify what would best promote this patient’s well-being in this particular context.^9^ It is the patient who is privy to their own values and to their particular life circumstances, relationships and position in society. However, sometimes their values conflict and sometimes their values should change, for example, a person who autonomously desires to abuse their partner. According to the liberal rationalist model, doctors and patients are engaged in a joint journey to answer the question: what should the patient do in this circumstance?

Productive engagement in a decision-making process can only emerge if the clinician is willing to ‘accept’, as a starting point, that the transgender individual’s claims have validity, if only insofar as they reflect their current best understanding of their predicament and their belief that it is the body that needs to change in order to improve well-being. This point may seem self-evident. However, historically, transgender individuals have felt that their claims have been discounted outright and that they have been the object of epistemic injustice.^10^ This has caused distress and added to the significant problems and stigma with which transgender individuals have had to contend.

An essential component of an ethical approach to this kind of decision-making process, we suggest, should be to ‘accept’ that the transgender individual has a unique perspective on what can make a positive difference to their predicament.^11 12^ Despite significant changes in the training of healthcare professionals towards shared-decision making and away from paternalism, there remains a risk that the patient’s account of what troubles them and/or what they need, is not regarded as having the same status as the clinician’s account. This is because, for example, the patient may be considered to be deluding themselves about the nature of the problem and/or about what will enhance their well-being.

It is against this background that current activism by the transgender community has managed to secure the adoption of a ‘gender affirmative’ approach within healthcare. In the first restricted sense of ‘to accept’, this change in practice can only be considered to be a positive development that protects individuals from the harms of epistemic injustice. However, gender affirmative care has recently been interpreted by influential sections of the transgender community as proscribing ‘questioning’ of any kind of the person’s stated gender and what will help them. This type of ‘acceptance’ is thus an altogether different proposition that makes assumptions deserving scrutiny, to which we will now turn.

It is worth noting that Mill was the champion of liberty and respect for autonomy, which he called individuality. He argued that we should each pursue our own original ‘existence’ and ‘the free development of individuality is one of the leading essentials of well-being’.^13^ Each person has ‘privileged access’ into their own nature and circumstances. Crucially, Mill realised that people are also corrigible about what is best for themselves. In the end, patients may have the right to choose a particular course of action which is not the best, out of respect for autonomy:

‘If a person possesses any tolerable amount of common sense and experience, his own mode of laying out his existence is the best, not because it is the best in itself, but because it is his own mode.’^13^

However, this should not short circuit a joint journey to attempt to identify what is genuinely in the person’s best interests. According to the liberal rationalist model, doctors and patients should walk this path together, even if in the end the patient departs to choose a legitimate option which is not best for him or her.

## Well-being and medical transitioning

The transgender individual, as defined here, typically seeks medical interventions that will allow them to realign the natal body with the gender of identification in order to reduce distress and so enhance well-being. The notion of well-being has been foregrounded in considerations about whether medical interventions are better overall for the transgender individual. Well-being has become an important conceptual currency in ethical debates. However, there is no general agreement about constitutes well-being as reflected in the different accounts of well-being (hedonistic, desire-satisfaction, objective list theories).^6^ More recently, a composite welfarist view of well-being has been proposed which includes hedonistic, desire fulfilment and objective elements.^6 14^ We draw on this latter conceptualisation because (A) it challenges us to consider that well-being depends on the values and interests of the individual and this resonates with the arguments put forward in debates by transgender individuals and (B) it helpfully places at its centre pluralism about value. This approach to well-being most clearly exposes the tension between the authority invested in medical and psychological ‘expertise’ about what makes a life go well and the individual’s right to choose what they consider to be in their best interests. It also gives considerable weight to the individual’s own desires and evaluations of their own interests.

A fundamental principle of bioethics is that patients should be offered interventions that are in their best interests. This is no longer restricted to offering treatments that will cure a medical or psychological condition, but now extends to include interventions that will enhance the psychological and/or social well-being of the person.^14^ This shift invites us to consider whether an intervention such as medical transitioning is, all things considered, better for a person if the psychosocial benefits outweigh the physical harms (e.g., of surgery) thereby enhancing that person’s well-being.

People’s values are central to the welfarist model. Values are personal and subjective, and sometimes idiosyncratic, hence decisions about what is best can only be considered on an individual basis. Gender identity is a case in point: how one feels in one’s body relative to one’s idiosyncratic experience of gender is a subjective state,^12^ such that it would be very difficult to assign an objective value to it. For some people, the risks associated with medical transitioning are deemed acceptable. From such a subjective vantage point (autonomy) and a composite welfarist account of well-being, we can make a strong case for ‘accepting’ the transgender person’s claims about what will make their life go better.

However, even if we take the individual’s privileged access and claims about their well-being, in the welfarist sense, as an essential starting point, we would still need to establish that the self-diagnosis is accurate such that the medical interventions are more likely to yield the anticipated benefits. If we take the example of the recent exponential rise in referrals of patients under 18 years who present for the first time to services postpuberty identifying as transgender, we have four strands of research and data that invite careful examination of whether the self-certification as transgender may reflect other psychological and/or societal problems:

1. Cross-sex identity in childhood is overwhelmingly predictive of homosexual orientation in adulthood. We must safeguard against conversion therapy by another name.^15^
2. Children with autistic spectrum disorders (ASD) are disproportionally represented in GID services.^16^ Given individuals with ASD’s preference for unequivocal answers, they may be more at risk of persevering with one definitive course of action instead of first exploring the complex emotional issues surrounding gender identity, especially during adolescence.
3. Natal girls are twice as likely to be referred to GID services.^17^ This urges us to investigate the role that social processes may play in the over-representation of girls.
4. The number of medically transitioned people coming forward now who regret transitioning is rising and has led to the creation in the UK of the Detransition Advocacy Network.^18^ These narratives indicate that some people consider that they had not been challenged enough to explore their reasons for wanting to transition. The recent legal case against the GID at the Tavistock Clinic is a case in point.

In the context of what has been termed an ‘epidemic’ in transgender identification,^19^ doctors have two moral duties: (1) to be wary of the moral panic currently surrounding the rise in transgender identification and ensure that this does not undermine the rights of transgender individuals and (2) consider the intrapsychic, social and/or cultural pressures that may unconsciously influence individual choice about transitioning thereby undermining autonomy and consent. Just as it would not be permissible to perform a mastectomy on someone who self-diagnosed as having breast cancer but in fact had bowel cancer, sex reassignment surgery, for example, on someone who self-identifies as transgender but may be suffering from an inability to accept that they are homosexual, would not be permissible.

It is contestable whether medicine should be promoting autonomy when it is against best interests. For the sake of argument, we will grant it should, but nonetheless what we want may not be an expression of our autonomy. At any rate it is important to work with the patient to first identify what is in their best interests.

## Autonomy, identity and the unconscious

We risk collateral harms if we treat all self-certified transgender people as a homogeneous group. It is clear that at least for some transgender individuals, medical transitioning is something that does not lead to an enhancement of well-being and is associated with later regret and/or continued levels of psychological distress. This suggests that conscious claims subjectively felt at the time to be reflective of the individual’s wishes may subsequently reveal that the impetus for medical transitioning was driven by other unconscious factors that undermined the individual’s autonomy and capacity to fully consent, and their ability to assess their own well-being.

Accounts by detransitioners that they have regretted the decision to transition provide pause for thought, but they cannot be assumed to be evidence that transitioning was therefore the wrong decision because people regret their decisions all the time. We cannot enter into a philosophical discussion about regret, but suffice to say that the possibility of potential future regret does not provide a strong argument for withholding treatment. The desire to prevent patient regret can be countered by appealing to the patient’s autonomy, which includes the right to make decisions that they might later regret. Not transitioning, we might argue, could also lead to regret. We suggest that although the possibility for future regret is a weak reason for not supporting a person’s wish to medically transition, inviting the patient to reflect on the possibility of later regret is an important part of a decision-making process.

Regret has a ‘complex temporal structure’.^20^ Here, we are addressing future regret that could result from unconscious forces that are operating at the time the decision is taken, as we will illustrate shortly with the (hypothetical) case of Sam. This needs to be distinguished from regret that could manifest further down the line due to a person’s change of preferences resulting from subsequent developmental experiences that inform self-understanding, identity and values retroactively. The process of reflection is in the service of supporting the identification of various conscious and unconscious drivers in order to provide access to a more comprehensive understanding of the self and of one’s values. This does not necessarily imply that the aim of psychotherapy is therefore primarily or only in the service of allowing us to identify what we ‘really wanted all along’. Rather it provides a broader perspective for assessing what is in one’s best interests at a given point in time.

What matters to patient autonomy is the integrity of the decision-making process with respect to medical transitioning so that the patient’s decisions reflect their core values, desires and preferences. This requires a broadened view of how we conceptualise autonomy and well-being that includes the role played by unconscious factors in decision making.

Autonomy has been variously defined and it is beyond the scope of this paper to review this vast literature. Nevertheless, several definitions emphasise two features of autonomy that we will focus on and examine through a psychoanalytic lens: ‘understanding’ and the ‘absence of controlling influences’^21^ and these have bearing for determination of interests.

### A psychoanalytic view of ‘understanding’ and of ‘absence of controlling influences’

Decisions about medical transitioning require access to factual data about the risks associated with these interventions and an understanding of the personal relevance of these risks for a given individual. ‘Understanding’, as we use the term here, is not restricted to cognitive understanding of medical facts, but we suggest that it should also involve self-understanding. This requires, as Freud put it, learning ‘first to know yourself’.^22^ This amounts to more than an intellectual understanding. Self-understanding, as conceptualised psychoanalytically, is about reducing the absence of controlling influences through broadening the scope of our awareness of the unconscious determinants of our decisions. By ‘controlling influences’ we have in mind, for example, the impact of external family and/or cultural pressures that are implicitly operating on our minds. We also consider the control exerted unconsciously by repetitive patterns in relationships with others and in relation to one’s ‘self’ that dominate an individual due to their life experiences and psychic defence structures. We will illustrate this shortly through consideration of Sam’s case.

What is meant by ‘self-understanding’ in psychoanalysis rests on some key assumptions about the mind that we will outline, but not defend, in this paper (for a fuller discussion see^23^):

1. We have a conscious as well as an unconscious mental life. Meaning systems thus include both conscious (i.e., verbalisable) and unconscious aspects of experience.
2. We all have a developmental history and a current life: both need to be understood if we are to understand ourselves and our decisions.
3. Our developmental history is relevant to self-understanding because our early attachments contribute to relationship templates that are often implicit (i.e., they operate without our conscious awareness) and that continue to shape behaviour in the present through the implicit activation of expectations of the self and of others. This ‘internal world’ of relationships gives texture and colour to each new situation that we encounter in the present: meanings and unconscious fantasies shape behaviour, thinking and feeling, and hence our decisions, whether or not they are the originators of the behaviour, thought or feeling.

Self-consciousness is considered a distinctive feature of human beings (and some ‘higher’ non-human animals) and is a central requirement for autonomy. We suggest that acknowledging the influence of unconscious mentation is also important. In considering the unconscious we have in mind not only the much-rehearsed question of unconscious brain processes that are precursors to an experienced choice^24^, but also how a significant proportion of our emotional reactions is controlled by automatic, unconscious structures, bypassing consciousness altogether.^25^ Research suggests that the same principles that apply to cognition operate with unconscious (implicit) affective and motivational processes as well. The so-called cognitive unconscious^26^ is now recognised to be the cognitive–affective–motivational unconscious.^27 28^

Research on unconscious affect provides persuasive evidence that we can feel things without conscious awareness that we feel them and that we can act on feelings of which we are unaware.^29 30^ Studies of subliminal perception, implicit cognition and directed forgetting have all shown how emotion processing can bypass the cortex and can proceed without conscious awareness.^29–33^ Classically conditioned emotional responses (e.g., expectations, preferences, desires) constitute the affective colouring of our lives. They orient us unconsciously to aspects of our environment and to particular types of relationships and inform the decisions we make. Often, there is no conscious memory connected with this learning. This makes it possible for emotionally charged schemas to be repeated without the mediation of consciousness.

Our perceptual system has evolved in response to the need to perceive not only accurately but also speedily. The brain has thus developed a split perceptual system.^29^ The slower perceptual system involves the cortex and includes conscious awareness. This system allows for more detailed information to be gathered, which in turn, helps us to inhibit responses and initiate alternative behaviours. The other system ‘‘fast-tracks’’ perception bypassing the cortex. This system does not involve any conscious awareness. The problem with the ‘‘fast-track’’ system is that it does not allow for a more fine-grained appraisal of what we are perceiving and responding to: it is not reflective. This means that when this system is activated, past experiences run a greater chance of impacting current experience though associative connections that are not reflected on, even when these may not be relevant or helpful to the immediate present. Current situations that are emotionally arousing, and where the capacity to reflect on experience is diminished by internal and/or external factors, are more susceptible to being fast-tracked. Risks to autonomy are posed when the decisions we make under these conditions are further fast-tracked by medical protocols that don’t do justice to the complexity of the unconscious factors that may be driving decisions. This is especially so when the decisions concern the subjective experience of identity, as we will now illustrate through a case study. The case is fictional and does not relate the history of any patient(s). However, the issues have been informed by the clinical psychoanalytic work by one of us (AL).

Sam, a natal girl, decided to transition from female to male during middle adolescence. The parents wholeheartedly supported this decision. They said that Sam had always been ‘tomboyish’. They were concerned about how depressed he had become, which the parents linked to body dysphoria. Sam scoured the internet for information about his difficulties and found solace in the narratives he read on trans websites. Following three consultations with a specialist GID, Sam was placed on cross sex hormones and changed his name.

At first Sam’s mood improved. He felt very connected to the trans community that he met online, and this eased some of his isolation. However, a year after starting cross-sex hormones, Sam took a serious overdose and was admitted to an in-patient facility. It was only at this point that he was referred for psychological help.

During the process of psychotherapy that ensued, it quickly emerged that Sam’s much older brother had died unexpectedly when Sam was still a very young child. The brother had been a strong presence in the family and Sam thought that his mother preferred him. He felt that his mother had never really recovered from this tragic loss. He conveyed how he had tried hard to please her as best he could and how upset he was when he felt unable to assuage her loss.

As a younger child, Sam did not recall wanting to be a boy, but he was clear that he had taken a strong interest in more typically ‘male’ sports (like Sam knew that the brother had done) and preferred playing with boys, which had alienated him from the girls at school. The parents had told Sam that he had expressed a strong wish to have been born a boy when he was at primary school. Sam had no recollection of saying this to them, but at the start of therapy he was nevertheless adamant that this was how he had always felt.

When Sam eventually decided that he wanted to transition, the parents were very supportive. Sam recalled feeling closer to them than ever before. He had the impression that they were happier overall, especially his mother. Over time in the therapy, it became clear that, as far as Sam was concerned, the decision to transition had facilitated a closeness between him and his mother that he had always longed for.

The parents appeared to have been very invested in Sam’s decision, even hurrying the process of starting hormones and attributed the suicide attempt exclusively to Sam’s gender dysphoria. The suicide attempt accelerated their belief that Sam should undergo sex reassignment surgery at the earliest opportunity. Yet Sam’s own account of the events leading to the suicide attempt did not appear to be evidently connected to gender dysphoria. Rather, the suicide attempt seemed to have been triggered by Sam’s feelings of distress and anger after the parents missed an important celebratory event in Sam’s life because it coincided with the anniversary of his brother’s death, which they always marked, and around which Sam often felt very uncomfortable.

The brother’s ‘ghost’ dominated the family’s collective psyche, for example, the brother’s bedroom had been preserved and Sam was often compared with his brother. Sam had very little memory of his brother and at times he resented the brother’s ongoing presence and preference in the mother’s mind. Sam was tortured by guilt that he had survived his brother as well as guilt about his resentment towards him for taking up the parents’ mental space which Sam wanted to claim for himself.

Sam had grown up in his brother’s shadow engulfed by the parents’ unresolved grief. In the context of therapy, Sam came to understand that the only way that he had felt he could be ‘seen’ by his parents was to effectively become their ‘son’ through transitioning. Yet, despite starting medical transitioning, Sam was soon confronted with the actual impossibility of replacing his brother. This became painfully clear to him when his parents cancelled their attendance at a celebratory event for Sam because it coincided with the anniversary of his brother’s death. Sam’s suicide attempt was another way in which he used his body to recall his parents’ attention to his existence and needs. But their misreading, as it were, of why he had tried to kill himself, left Sam feeling alienated from them and confused about whether he should seek sex reassignment surgery.

We will not discuss this case further but suffice to say that Sam’s trajectory following the suicide attempt was punctuated by a series of crises and a prolonged depressive state marked by confusion over his identity and uncertainty as to whether he had made the right decision to commence transitioning. As the family dynamics were consciously articulated, gender identity receded in Sam’s mind as either the problem or the solution and the focus turned instead to Sam’s distress and grievance towards his parents for what he had experienced as their neglect of his needs. Awareness of how the unresolved family grief and his own longing to be the replacement son had informed his transgender identification enabled him to make informed choices about the best next steps for him.

### Unconscious aspects of identity

A central contribution by psychoanalysis to discussions about identity is that it destabilises any such notions by introducing the role of the unconscious and the ‘speciousness’ of identity itself.^34^ Tying down identity primarily to social processes and conscious choice denudes the notion of its essentially conflictual nature and of its intimate connection with desire and unconscious fantasy.

The sexed body, social gender and sexuality (i.e., desire) are all constitutive of identity. Gender identity is not an indicator of sexual orientation and both are nowadays conceptualised as independent of sexed bodies. The value of a psychoanalytic perspective on gender and sexuality is that it reveals that the relationship between a body part and its sexual function or its gendered significance is at best one of ‘lightly tethered consonance rather than a rigidly shackled indexical mapping’.^35^

The psychic investment that we have in our body, its form and appearance is key to understanding the subjective experience of embodiment and hence needs to be reflected on in clinical and theoretical discussions of identity. The body is the primary site of inscription and meaning arising from external forces as well as internal, unconscious ones. We suggest that understanding the breadth of meaning and function that is subsumed under ‘transgender’ as an identity referent is helped if we think not only in terms of societal ‘gender’ ascriptions but also in terms of the subjective experience of embodiment, of the body’s unconscious identifications and hence the psychic function of the modification of the body.^36^ This is important because the representation that we have of our bodies in our minds is profoundly shaped by the projections of others—often early attachment figures—onto our bodies. In order to protect autonomy, it is important to disaggregate the beliefs and desires that belong to the individual wishing to transition from those that they think belong to them but may in fact be better understood as serving other unconscious functions such as, for example, appeasing another person’s desires, as we have illustrated with Sam’s case, or that may be unconsciously felt by the individual to be preferable to other less preferable alternatives (e.g., the person whose homosexual orientation is felt to be more laden with difficulty than coming out as transgender).

The unconscious aspects of identity deserve consideration because our sense of who we are—of our identity—derives in part from our early developmental experiences. Attachment research provides a further strand of evidence lending support to how early relationships unconsciously shape the development of the mind and what is referred to in psychoanalysis as the ‘internal world’.^37^ The internal world consists of prototypic schemas involving invariant dimensions of early affectively charged relationships (e.g., experiences of union and separation). In early life, heightened affective exchanges are psychically organising: they allow the baby to categorise and expect similar experiences. For example, a ‘negative’ experience of rejection may be internalised as a working model of an ‘ugly’-self-relating-to-a humiliating-other’. Once learnt, a relational working model unconsciously operates like a template for interpreting later events in a similar way; that is, it generalises. External relationships at any stage of the lifecycle may then trigger the affect associated with particular relationship constellations and the associated relational fantasies (e.g., ‘If I get close to another person, they will see my ugly self and humiliate me, so it’s best to keep others at a distance’). These mental representations of ‘self-affectively-interacting-with-other’ therefore contain both conscious and non-conscious cognitive and affective components deriving from significant interpersonal experiences with key attachment figures. Although the experiences that contributed to these schemas remain for the most part inaccessible to conscious memory because they are procedurally unconscious (i.e., are not registered in autobiographical memory), they nevertheless structure how we think and feel about ourselves and about others in the present. This is why, even though we may not be able to recall early events, we nevertheless continue to organise the present according to developmental models. In considering the significance of unconscious mentation to autonomous choice, we are therefore interested in the relevance of an unconscious internal world of relationships coloured by intense affects that influences our behaviour and decisions.

### The ethical significance of reflective spaces for supporting autonomy

Our current understanding of the unconscious mind points to an important fact: ‘*‘*What is most meaningful in life is not necessarily encoded in words’’.^38^ This, we suggest, has important implications for how we might understand autonomy, well-being and consent. If, as psychoanalysis proposes, (1) we are formed by early relational experiences for which we have no declarative memories, (2) our present behaviour is informed unconsciously by repetitive relational and affective patterns that exert an impact on our experience of our self, of our desires and of the decisions we make, and if we are not self-reflectively aware of these patterns, then consent to medical interventions sought in order to instantiate physically our subjectively felt sense of who we are is potentially undermined.

Autonomy, we suggest, is enhanced by consideration of the implicit relational and affective templates that shape our experience of who we are and who we may feel that we ought to be and that underpins our decisions. Understanding of these patterns requires time and dialogue with others who can help us to decipher what is unconscious. The reflective space that psychoanalytically informed psychotherapy provides is the example, par excellence, of the kind of constructive dialogue that can support autonomy in decision-making due to its attention to conscious as well as unconscious processes. There is now accumulating evidence that psychoanalytic psychotherapy is an effective intervention for a range of mental health problems.^39 40^

A person is deemed to have the capacity to rationally choose iff she has at her disposal knowledge of all the facts relevant to the decision and the possible consequences for her of the potential decisions.^5^ We further propose that the condition of ‘being in possession of all available relevant facts’^5^ should include the specification that these ‘facts’ also involve consideration of possible unconscious drivers (e.g., wishes or unprocessed traumas). Once these unconscious influences become objects of conscious reflection, we can take steps to reduce or accommodate their effect on our behaviour and decisions. Although unconscious factors pose a threat to autonomy, the more we understand the extent to which we are unconsciously motivated and influenced, the better equipped we are to function autonomously. Becoming conscious of an unconscious wish or motive involves experiencing that wish or motive as one’s own. This makes it possible for us to assess whether we want to cultivate it or ignore it.

Importantly, our values may be unconscious or inarticulated. What we truly most desire may be opaque to us. Not only will psychoanalysis reveal facts about us, but it can reveal the nature of our values. This is important for most contemporary models of the doctor–patient relationship. It sits most comfortably with the liberal rationalist model which aims at normative dialogue to arrive at both the relevant facts for the patient but also what the patient should value. It is also important to shared decision-making where the patient must provide the values: those values may be unconscious. This might seem to provide a kind of ‘master self’ view: there is a rational self with values that the unconscious self is undermining, and we need to bring the motivations and influences of the unconscious self to the attention of the master self who will decide what to do with them. But the master self is also the result of these influences—there is a bootstrapping problem. The very values we have are the product of these influences and indeed may be unconscious.

Acknowledging the machinations of the unconscious reminds us of the limits to autonomy. Does this mean therefore that autonomy is an illusory state forever subverted by the unconscious? This requires some qualification. Autonomy is not an ‘all or nothing’ state. We are not making the absolute claim that engagement in a psychoanalytic process ensures that we reach at some point a fully autonomous state where we are no longer informed by unconscious drivers. The unconscious is a mental structure that continues to potentially exert its influence on our conscious decisions. Like a physical muscle that, if left unexercised, quickly reverts to its former state, our engagement with understanding the unconscious is not a once and for all process: it requires the regular exercise of this psychic muscle (i.e., ongoing self-reflection). If we exercise this psychic muscle, we stand to reap some benefits: we have an enhanced opportunity of identifying some of the pressures on our decisions that arise out of unconscious drivers and we can bring these more under conscious deliberation. The ‘benefits’ do not include the eradication of the unconscious—that is, an impossibility. To this extent, claims about autonomy in relation to the unconscious are always relative. We are making the more modest claim here that exploration of unconscious drivers supports the expansion of the range of autonomous functioning, not that it makes us autonomous in an absolute sense.

So far, we have argued that our conscious choices may be limited by powerful a priori motivations that can only assist us in decision-making through second-order reflection and evaluation. This process requires time. Time for self-reflection is not only important psychically, but it is also ethically significant because it supports autonomous decisionmaking given that the elaboration of unconscious meaning emerges piecemeal over time. This is one reason why unqualified acceptance of conscious claims can lead to unintended harms.

Clearly not all individuals who express the wish to transition are motivated by underlying psychological problems unconnected to gender identity as such, as was the case for Sam. We are therefore not suggesting that requests for transitioning should be regarded suspiciously. Rather, we are suggesting that cases such as Sam’s caution us to consider how our medical protocols can best safeguard spaces for reflection should what appears on the surface to be a well-thought-out decision be just the tip of an iceberg of meaning and psychological needs that will not be successfully met by transitioning.

Sam’s case underscores the complexity of assessing autonomy and one’s own assessment of one’s interests when we factor in systemic and intrapsychic unconscious pressures on self-perception and identity development. Sam appeared to have been driven primarily by a wish to claim a space in his parents’ mind (especially his mother) that he felt was occupied by his deceased brother. He unconsciously resolved to do this by identifying as a boy and then asking to transition so that he could replace his brother. Even though the family was not consciously coercing Sam to change gender, it could be argued that there was an unconscious investment in supporting the gender transition so as to reclaim their lost son. Systemic pressures can thus potentially function to enforce conformity to unconscious needs that act against the person’s best interests.

Sam’s case can, and indeed should, be countered by cases that reveal that transitioning was, all things considered, better for the person. Such cases are important reminders that medical transitioning can enhance well-being and be an expression of autonomy. However, the fact that this can be the outcome of transitioning for some is not an argument against a thorough exploration of the motivations for wanting to transition so as to ensure that the decisions taken can be said to be substantially autonomous and substantially independent from controlling forces such that the person ‘…is free of controls exerted either by external sources or by internal states that rob the person of self-directedness’.^21^ Proscribing such exploration sets up a climate that can unwittingly promote potential harm rather than reduce it, as Sam’s example illustrates. Sam’s case illustrates the operation of the relationship between external and internal pressures on decision making about transitioning. His family was driven by their own grief to pressurise Sam into transitioning. This external pressure, however, is not recognised as such consciously by Sam. It is unreflectively internalised and takes on a life of its own in the idiosyncratic prerogatives of his internal world, for example, his own unconscious longing to be loved by his parents and to replace his brother and the guilt this provokes.

Affirmation of that which is not in fact the truth is undermining of autonomy and likely to frustrate interests. As Beauchamp and Childress^21^ underline, if a patient holds a false belief about their condition, their decision about treatment is compromised. Full autonomy requires not only presentation of medical facts but that the patient holds rational beliefs about their condition, the possible interventions, their risks and benefits, but importantly themselves and their psychic nature.^5^ A decision to transition cannot be an instantiation of autonomy if it is based on a false narrative that prevents the person from accessing the help and resources appropriate to the state that actually undermines their well-being. The individual’s current wishes, no matter how strongly felt, are not always a reliable indicator of what will enhance well-being. By contrast, engagement over time in a process of psychotherapy may open up new possibilities for greater autonomy and well-being than that potentially afforded by (premature) transitioning.

To be genuinely autonomous is to understand oneself both at the conscious and unconscious level. That said, it is important to not allow the re-entry of old-style paternalism and the imputation of desires and values at an unconscious level on the patient. It must be the patient who does the work and identifies these unconscious properties, with the assistance of the therapist. It is also important that patients can choose options which are ultimately less than the best. Mill described experiments in living as the best way to achieve individuality (autonomy). Maybe the best we can hope for is to live in different ways, reversibly, before we choose a final path. Freedom is, in part, taking responsibility for living with your choices.

## Conclusion: what should gender affirmative care ‘accept’?

We have argued that if by ‘accepting’ the transgender individual’s claims about what will make their life go better, we mean that we do so unreservedly or without the provision of a reflective space in which both conscious and unconscious motivating factors can be safely explored, then we should not accept their claims prima facie. We have suggested that we have ethical grounds to advocate a respectful, collaborative, and inquisitive approach so as to ensure that the desire to medically transition can be said to be autonomous. We suggest that the argument holds for any transgender individual irrespective of age. Indeed, our arguments apply to autonomy and well-being in medicine generally.

If we accept that a decision to medically transition needs to be at least substantially autonomous and substantially independent from controlling forces,^21^ then such a decision should only be taken after a period of exploration given the potential controlling influences (internal and external) that may bear on this particular decision. Unqualified acceptance can reduce the individual’s options and autonomy if it deprives them of an opportunity to explore the possible unconscious meaning of their transgender identification which, in turn, might lead them to consider options other than medical transitioning. This will likely enhance their long term well-being. Reflective spaces, such as those provided by psychotherapy, allow unconscious processes to be brought to (conscious) attention, potentially increasing the scope of reflective control. We have argued that this increases the range of autonomous functioning rather than claiming that it makes the individual free of the unconscious in an absolute sense.

We must distinguish the laudable aims of avoiding prejudice and protecting the right of the transgender individual to self-determination and autonomous choice about their well-being from the unintended consequences of ‘affirmative gender care’. An inquisitive approach should guard against the possibility of perpetrating epistemic injustice, but inquisitive self-exploration can also enhance autonomous choice, thereby promoting well-being that reflects the values and interests of the individual.

Such an inquisitive approach is consistent with most—and demanded by some—contemporary non-paternalistic models of the doctor–patient relationship. If transitioning is a reasonable option for the patient, the patient may in the end opt for it, even if it is less than the best course of action. In this sense, autonomy may trump interests. But the patient deserves, and has a right to, the journey to identify what is most likely to promote their well-being.

While we have concentrated on gender affirming care by way of an example because this is a current concern that generates moral panic, similar arguments apply to many areas of medicine that involve controversial value judgements,^7^ such as sterilisation, IVF, enhancement, especially cosmetic enhancement, the use of relationship therapy, and ‘love drugs’.^41^
